# The physiological and pathological properties of Mead acid, an endogenous multifunctional n-9 polyunsaturated fatty acid

**DOI:** 10.1186/s12944-023-01937-6

**Published:** 2023-10-14

**Authors:** Hiroshi Kawashima, Katsuhiko Yoshizawa

**Affiliations:** 1 Research Institute, Suntory Global Innovation Center Ltd, Seika, Kyoto Japan; 2 Department of Innovative Food Sciences, School of Food Sciences and Nutrition, Mukogawa Womenʼs University, Nishinomiya, Hyogo Japan

**Keywords:** Mead acid, Eicosatrienoic acid, n-9, Arachidonic acid, Essential fatty acid, Polyunsaturated fatty acid

## Abstract

Mead acid (MA, 5,8,11-eicosatrienoic acid) is an n-9 polyunsaturated fatty acid (PUFA) and a marker of essential fatty acid deficiency, but nonetheless generally draws little attention. MA is distributed in various normal tissues and can be converted to several specific lipid mediators by lipoxygenase and cyclooxygenase. Recent pathological and epidemiological studies on MA raise the possibility of its effects on inflammation, cancer, dermatitis and cystic fibrosis, suggesting it is an endogenous multifunctional PUFA. This review summarizes the biosynthesis, presence, metabolism and physiological roles of MA and its relation to various diseases, as well as the significance of MA in PUFA metabolism.

## Introduction

**Fig. 1:**
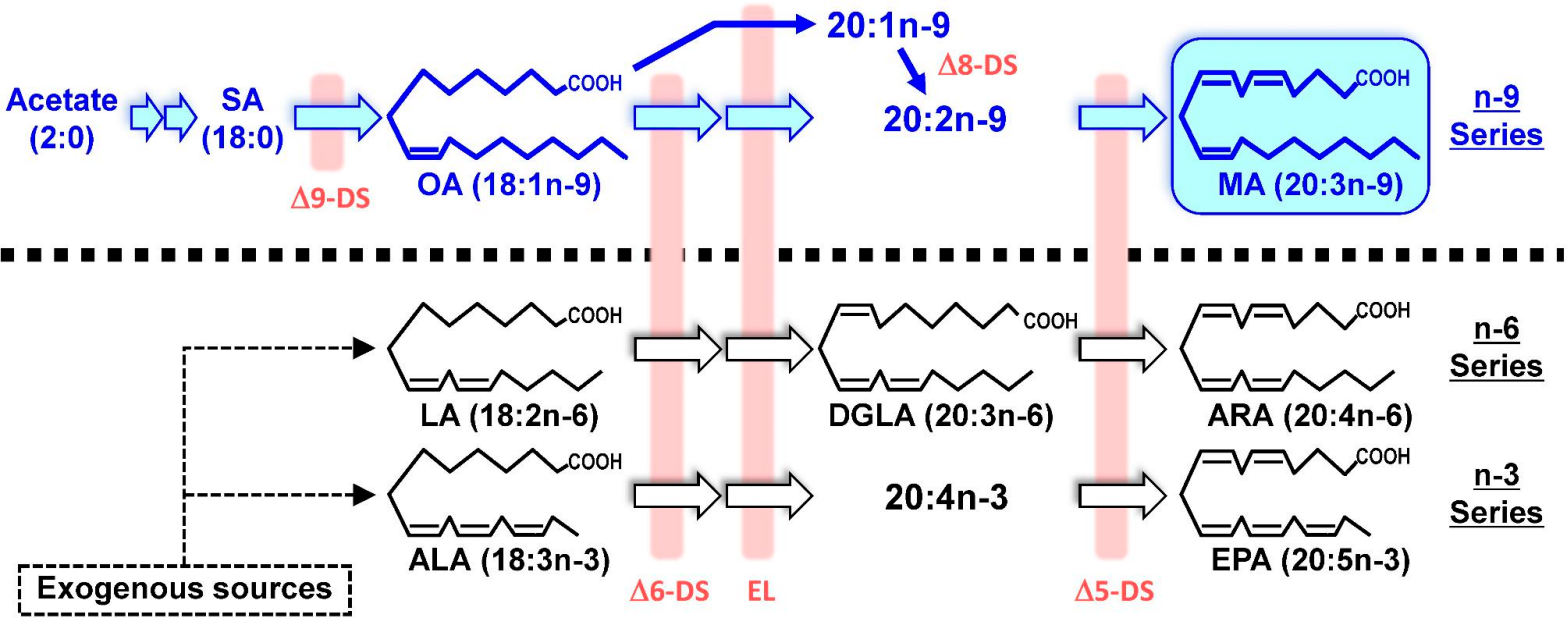
Biosynthetic pathway of mead acid and other polyunsaturated fatty acids. ALA, α-linolenic acid; ARA, arachidonic acid; DGLA, dihomo-γ-linolenic acid; DS, desaturase; EL, elongase; EPA, eicosapentaenoic acid; LA, linoleic acid; MA, mead acid; SA, stearic acid

Mead acid (MA, 5,8,11-eicosatrienoic acid) is an n-9 polyunsaturated fatty acid (PUFA). MA was first identified by Mead and Slaton [1] in rats fed a fat-deficient diet and was determined to be derived from oleic acid [2]. Essential fatty acid (EFA) deficiency induces skin rash, alopecia, growth disorders and reproductive abnormalities [3, 4], accompanied by the appearance of MA in the blood. In EFA deficiency, MA derived from oleic acid appears to be synthesized rather than arachidonic acid (ARA) from linoleic acid (LA) (Fig. 1). MA is widely used as a marker of EFA deficiency; for example, the ratios of MA/ARA [5] and trienoic/tetraenoic acids are used to identify EFA deficiency [6–8]. Recent findings suggest that MA plays an important role as an endogenous PUFA, and that it is related to various diseases, such as inflammation, cancer, dermatitis and cystic fibrosis. Although these interesting studies have been reported individually, there has not yet existed a comprehensive overview of all available information about MA. The aim of this review is to provide an overview of the impact of MA in PUFA metabolism, the biosynthesis, presence and physiological roles of MA, and its relation to various diseases.

### Biosynthesis and presence of MA

Most typical PUFAs belong to the n-6 or n-3 series and are derived from exogenous linoleic acid (LA) or α-linolenic acid (ALA), respectively. LA and ALA are converted physiologically to arachidonic acid (ARA) and eicosapentaenoic acid (EPA), respectively, by Δ6-desaturase, elongase and Δ5-desaturase. MA is synthesized from oleic acid (OA) in the n-9 series *via* the same enzyme system as for the n-6 and n-3 series, although Ichi et al. suggested another pathway *via* 20:1n-9 and 20:2n-9 [9].

MA has two distinct characteristics. First, MA is *de novo* synthesized from other carbon sources, such as acetate and sugars. MA is considered an endogenous PUFA, although all n-6 and n-3 PUFAs are derived from exogenous plant-derived PUFAs. Second, the amounts of MA and other n-9 PUFAs present in the body are small whereas ARA and EPA are abundant. Δ6-Desaturase, the rate-limiting enzyme in PUFA synthesis, has a much higher affinity for ALA and LA than for OA. Therefore, Δ6-desaturation of OA is highly suppressed in the presence of LA and ALA [10]. MA formation requires a marked decrease in both LA and ALA systemically or topically. Conversely, most tissues that synthesize ARA can potentially synthesize MA from endogenous OA.

MA is present systemically in EFA deficient animals, but is found in the plasma, blood vessels, liver, cartilage and eye lens of non-EFA-deficient animals. MA is also found in cultured cells and filamentous fungi [11–20] (Table 1). A small amount of MA is present in the major tissues of healthy adult humans, including in plasma (0.16%) [11], serum (0.24%) [13], blood vessels (0.1%) [15] and the liver (0.2%) [16]. Because MA is present at low concentrations, it is often overlooked, but this small amount of MA is distributed widely in various tissues. OA is an abundant fatty acid in various tissues and is a poor substrate for Δ6-desaturase. MA is found in muscle (1.42%) [14], spleen (0.2%) [16], brain (not quantified) [21], growth plate cartilage (4.67% and > 3%) [14, 22], cortical bone (0.36%) [14] and cord blood mononuclear cells from term (0.27%) and preterm (0.11%) [23], in addition to the tissues listed in Table 1.

Relatively large amounts of MA are detected in fetal tissues, including plasma (0.44%) [11], blood vessels (2.4%) [15] and the liver (0.4%) [16] (Table 1). Although ARA levels are much higher than those of MA in fetal tissues, LA is present at a low concentration of < 10%, which is less than one third the concentration in corresponding adult tissues, suggesting that fetuses are in a state of systemic and mild EFA deficiency. It supports that EFA intake by infants from breast milk or infant formula is necessary [24, 25]. For example, the MA content of fetal or infant cartilage is 2.0% (human), 3.0% (sheep) [16], 5.12% (calf) and 8.11% (pig), and is as high as 16.98% in 8–10 week-old chickens [14]. It is unclear why the MA concentration is so high in young cartilage but this may be due to cartilage being an avascular tissue, making it difficult to incorporate sufficient amounts of exogenous LA and ALA during development. The lens is another avascular tissue and contains 3.19% MA in near-term calves [14]. The effects of MA on angiogenesis are shown as described below. Aside from cartilage, a large amount of MA was found in the liver of rats fed a peroxisome proliferator-activated receptor (PPAR) α agonist (> 6% in the triacylglycerol fraction) [12], possibly due to the induction of desaturases and elongases by the PPARα agonist.

Importantly, cultured cells can contain considerable levels of MA (3.8% and 0.5%) [18, 19]. Several papers report quantifiable amounts of MA in cultured cells, and MA 8 be present in many cell cultures. Recently Okuno et al. used LC-MS/MS to show that the concentrations of polar lipids containing MA as acyl moiety increased in RAW 264.7 cells with increasing culture time, and that eicosanoid production was altered [26]. In pseudo-EFA deficiency, MA may tend to accumulate, for example, in rapidly growing cells whose capacity for EFA incorporation is low and for which the concentration of EFAs in the medium is limited. Potential EFA deficiency in cultured cells requires further study.

MA is also produced by filamentous fungi. A wild-type strain of the fungus *Mortierella alpina* produces an ARA-containing oil, and Δ12 desaturase-deficient mutants have been obtained [20]. These deficient strains synthesize MA (25.5%) instead of ARA (Table 1), similar to EFA-deficiency in mammals, and MA-containing oil accumulates in the cells [27, 28]. This MA-containing microbial oil is an alternative source of MA in addition to MA synthesized chemically [29, 30], and has been used in the pharmacological studies described below.

**Table 1:** Existence of MA in organisms other than systemic essential fatty acid deficiency

Tissue or cell	Organism	Characteristics	Fraction^a^	Fatty acids (%)^b^						Ref.
				C20-22 PUFA				C18 PUFA		
				MA	ARA	EPA	DHA	OA	LA	
				20:3n-9	20:4n-6	20:5n-3	22:6n-3	18:1n-9	18:2n-6	
Plasma	Human	Neonate, venous umbilical plasma	PL	0.44	17.07	0.38	6.74	nd^c^	6.40	[11]
		Pregnant mother	PL	0.16	7.93	0.64	4.79	nd	20.81	
	Rat	PPARa agonist-fed	TL	> 3^d^	< 5^d^	nd	nd	nd	nd	[12]
		Olive oil-fed	TL	0.7	6.4	nd	nd	nd	nd	
Serum	Human	18–33 years old	PL	0.24	9.25	0.79	2.93	11.95	21.57	[13]
	Calf	Near-term, articular	Polar L	0.46	7.79	0.58	3.85	15.79	2.00	[14]
	Chicken	8–10 weeks old, articular	Polar L	0.47	26.01	nd	nd	6.37	11.71	
Blood vessel	Human	Neonate, umbilical arteries	PL	2.53	12.35	0.03	5.48	nd	1.04	[11]
	Human	Neonate, umbilical arteries	PL	2.4	12.0	0.05	4.7	13.5	1.7	[15]
		Adult, colonic arteries	PL	0.1	20.3	0.3	3.2	13.4	5.1	
Liver	Human	Fetal, 18–38 weeks of gestation	TL	0.4	22.9	0.2	7.3	11.9	6.6	[16]
		Adult, 37–90 years old	TL	0.2	12.2	0.8	2.4	12.9	14.6	
	Human	Fetal, 24 weeks of gestation	PL	0.70	18.42	0.45	4.39	12.13	4.59	[17]
	Rat	PPARa agonist-fed	TL	> 6^d^	< 16^d^	nd	nd	nd	nd	[12]
		Olive oil-fed	TL	1.8	14.2	nd	nd	nd	nd	
Cartilage	Human	Fetal, femoral head	TL	2.0	15.2	< 0.1	2.8	21.4	2.9	[16]
		Infant, femoral head	TL	1.5	11.0	< 0.1	1.1	22.3	6.1	
		Adult, femoral head	TL	< 0.1	9.1	< 0.1	0.8	13.5	8.9	
	Sheep	Fetal	TL	3.0	6.0	0.9	2.7	30.4	0.6	[16]
		Mature	TL	0.1	4.1	0.4	1.0	25.6	6.0	
	Calf	Near-term, articular	Polar L	5.12	5.13	0.40	1.66	29.61	1.49	[14]
	Pig	Newborn, articular	Polar L	8.11	6.53	0.34	1.22	23.14	1.21	
	Chicken	8–10 weeks old, articular	Polar L	16.98	9.42	nd	nd	24.51	4.78	
Lens	Calf	Near-term	Polar L	3.19	7.48	0.48	2.18	30.68	2.61	[14]
Cell line	Rat	PC12, adrenal pheochromocytoma	PE	3.8	8.8	0.3	1.9	14.5	1.7	[18]
	Human	T-24, bladder cancer	TL	0.5	6.3	3.6^e^		34.9	4.6	[19]
Mycelium	Fungus	*Mortierella alpina* M209-7	TL	25.5	< 0.5	< 0.5	< 0.5	37.0	< 0.5	[20]

^a^*PL* phospholipids, *TL* total lipids, *Polar L*, polar lipids

^b^weight % except for [12] (mol%). *MA* mead acid, *ARA* arachidonic acid, *EPA* eicosapentaenoic acid, *DHA* docosahexaenoic acid.

^c^not described

^d^approximate number read from Fig. 4 in [12]

^e^EPA + DHA

### MA metabolism and its effects on the fatty acid profile

MA is detected in organisms with EFA-deficiency but EFA-deficient organisms are not always suitable for understanding the exact metabolism of MA and its effects on the fatty acid profile because the simultaneous drastic decrease in EFAs, such as ARA and docosahexaenoic acid (DHA), in such organisms makes it difficult to study MA metabolism.

This was addressed by administering exogenous MA to rats or mice, for 3 to 8 weeks, which increased the MA concentration in the plasma, liver, spleen, peritoneal exudate cells [31–34], transplanted human breast cancer cells [35], mammary tissue [36], lens and retina [37]. A dose-dependent increase of MA was observed in the plasma, liver, spleen and peritoneal exudate cells [31] in which the percentage of MA in fatty acids was above 20% [31, 33, 35–37]. These results suggest that MA can be absorbed from the digestive tract and transferred to various tissues efficiently. MA was detected as acyl moiety of phospholipids, triglycerides and cholesterol esters in plasma [31], and that of phospholipids in the liver, spleen and peritoneal exudate cells [31–33]. In peritoneal exudate cells, MA was contained in phosphatidylcholine, phosphatidylethanolamine and phosphatidylinositol [33]. These findings suggest that the absorption and distribution of MA is widespread physiologically and is similar to that of other C20 PUFAs, such as ARA and EPA.

Most of the studies described above also detected docosatrienoic acid (DTA, 22:3 n-9), believed to be formed by the elongation of MA, similar to the C20 analogues, ARA and EPA. The DTA concentration was around 3–7% and was almost half that of MA in spleen and peritoneal exudate cells [31, 33, 34]. The presence of the further metabolites, tetracosatrienoic acid (24:3 n-9), tetracosatetraenoic acid (24:4 n-9) and docosatetraenoic acid (22:4 n-9), was not detected, although the homologous metabolites in the n-6 and n-3 series were well known, with docosapentaenoic acid (22:5 n-6) and DHA formed, respectively [38]. Metabolism through the C24 fatty acids of the n-9 series may be impeded, apart from the n-6 and n-3 series.

The above studies showed that the concentrations of other PUFAs, such as ARA, EPA and DHA, decreased due to their partial displacement by MA. The results suggest that MA competes with other long chain PUFAs in PUFA metabolism, and that the presence of MA is not solely due to the decrease in ARA in patients with EFA deficiency. The competition of MA with other PUFAs, especially ARA, is a main mechanism underlying its various physiological and pathological activities, as described below.

Careful attention is needed for estimating the effects of MA on the increase in MA concentration or the displacement of other PUFAs in tissues. In most cases, large amounts of fatty acids other than MA are contained in MA-containing oil itself [20, 27, 28] or as ingredients of diet or culture medium, and may interact with the effects of MA.

### Conversion of MA to lipid mediators

**Fig. 2:**
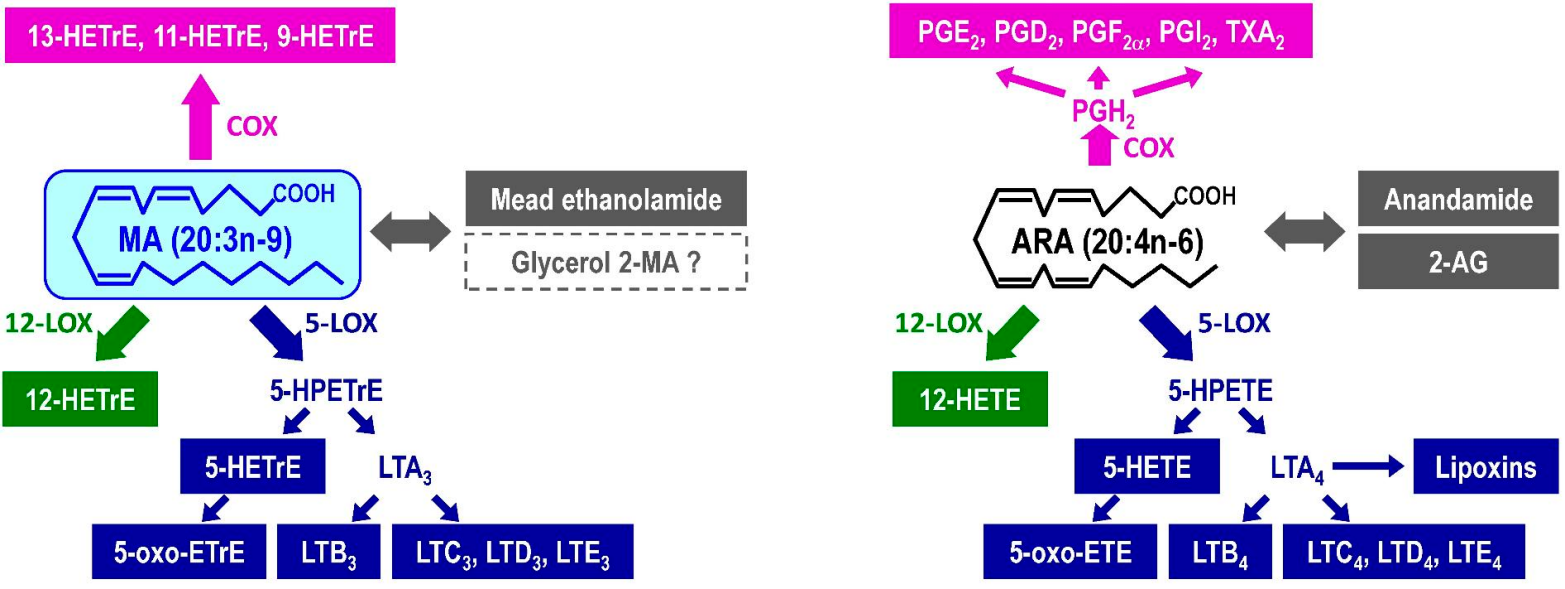
Correspondence relation of lipid mediators from MA (left) and ARA (right). AG, arachidonoyl glycerol; ARA, arachidonic acid; cox, cyclooxygenase; ETE, eicosatetraenoic acid; EtrE, eicosatrienoic acid; HETE, hydroxyeicosatetraenoic acid; HETrE, hydroxyeicosatrienoic acid; HPETE, hydroperoxyeicosatetraenoic acid; HPETrE, hydroperoxyeicosatrienoic acid; LOX, lipoxygenase; LT, leukotriene; MA, mead acid; PG, prostaglandin; TX, thromboxane

MA is now recognized to also be converted to various lipid mediators similar to those of from ARA (Fig. 2). We here review the current knowledge and explore avenues for further research into the roles of MA-derived lipid mediators in cardiovascular biology, carcinogenesis and many inflammatory diseases [39–41]. This conversion is due to MA having a structure similar to that of ARA and having identical double bonds at the 5, 8 and 11 positions. The properties of most of lipid mediators derived from MA remain unclear but may be related to the physiological and pharmacological activities of MA. It should, incidentally, be noted that it is important to distinguish MA from another eicosatrienoic acid, dihomo-γ-linolenic acid (DGLA), which has double bonds at the 8, 11 and 14 positions and is an n-6 PUFA (Fig. 1). DGLA has various unique activities and is metabolized to specific lipid mediators that differ from MA-derived mediators [42–44].

#### 5-Lipoxygenase (LOX)

Leukotriene (LT) is a major 5-LOX metabolite. LT derived from ARA is categorized as 4-series [41]. 5-LOX converts ARA to 5-hydroperoxyeicosatetraenoic acid (5-HPETE) and then to LTA_4_. LTA_4_ is conjugated with glutathione to form LTC_4_, which is sequentially converted to LTD_4_ and then LTE_4_. LTA_4_ can be also converted to LTB_4_ by LTA_4_ hydrolase. 5-HPETE can be converted to 5-hydroxyeicosatetraenoic acid (5-HETE) and 5-oxo-eicosatetraenoic acid (5-oxo-ETE).

Of the MA-derived lipid mediators, the 5-LOX metabolites have been the most intensively studied since the 1980s. Most ARA-derived 5-LOX metabolite analogues are formed from MA, i.e., the 3-series LT. Mouse mastocytoma cells were found to convert MA to LTC_3_ and LTD_3_ [45]. The distribution [46] and metabolism [47] of LTC_3_, and the conversion of LTD_3_ to LTE_3_ [48], were reported successively. LTA_3_ is a poor substrate for human neutrophil LTA_4_ hydrolase and a potent inhibitor of this enzyme [49], which may cause reduction of LTB_4_ as described below. A tyrosine residue (Y383) of LTA_4_ hydrolase attacks the conjugated triene epoxide of LTA_3_, resulting in the covalent attachment of LTA_3_ to LTA_4_ hydrolase [50].

LTB_3_ has been found in cultured cells [26], despite the fact that LTA_3_ inhibits LTA_4_ hydrolase as described above. LTB_3_ can displace [^3^ H]-LTB_4_ from both rat and human leukocyte membranes with the following affinity: LTB_4_ = LTB_3_ > LTB_5_ [51]. 5-oxo-ETE is a potent chemoattractant for neutrophils and eosinophils formed from ARA, and its actions are mediated by the oxoeicosanoid receptor. Similarly, 5-oxo-ETrE is formed from MA, and activates granulocytes with a potency similar to 5-oxo-ETE [52].

Lipoxins (LX) are pro-resolving lipid mediators formed from ARA by 5-LOX and 12/15-LOX [53]. Because MA lacks a Δ15 double bond, no LX-type mediators are likely formed from MA. LXA_4_ and LXB_4_ are produced upon incubating LTA_4_ with human platelets, but not upon incubation with LTA_3_ [54].

#### 12/15-LOX

12/15-LOX converts ARA into 8-HETE, 11-HETE, 12-HETE and 15-HETE [55, 56], and thus the corresponding HETrEs might be generated from MA. The HETrE 12-hydroxy-5,8,10-eicosatrienoic acid (12-HETrE) is formed from MA in human platelets [57] and exhibits prostaglandin (PG) E_2_-like biphasic effects on platelet aggregation. Other MA-derived HETrEs are likely catalyzed by 12/15-LOX but have not been reported.

#### Cyclooxygenase (COX)

COX converts ARA into PGH_2_, which has a characteristic five-membered ring, and then is metabolized further to various prostanoids. However, COX converts MA into several metabolites lacking a five-membered ring, and especially into 13-hydroxy-5,8,11-eicosatrienoic acid (13-HETrE) [58, 59]. 11-Hydroxy-5,8,12-eicosatrienoic acid (11-HETrE) and 9-hydroxy-5,7,11-eicosatrienoic acid (9-HETrE) are minor metabolites. COX-2, an inducible type of COX, also converts MA to 13-HETrE and 11-HETrE [60]. These HETrEs are formed by COX, not by LOX, but the conversion of MA by COX is slow compared with the conversion of ARA [60, 61]. In addition to these HETrEs, 8,11-dihydroxy-5,9,12-eicosatrienoic acid from MA was reported [58].

### Mead ethanolamide and glycerol 2-MA

Arachidonoylethanolamide and 2-arachidonoylglycerol (2-AG) are endogenous agonists of cannabinoid CB1 and CB2 receptors [62]. Mead ethanolamide is biosynthesized from MA in rat and human hippocampal membranes as efficiently as arachidonoylethanolamide is synthesized from ARA [63]. Chemically synthesized mead ethanolamide is equipotent to arachidonoylethanolamide as a competitor of the cannabinoid agonist CP55,940, and it binds to plasma membranes expressing the human CB1 receptor [63].

A principal pathway to 2-AG is the degradation of 2-arachidonoyl phospholipids to 2-arachidonoyl-diacylglycerol by phospholipase C, followed by deacylation to 2-AG by an *sn*-1-specific diacylglycerol lipase [63]. Phosphatidylinositol [26, 64] and phosphatidylcholine [64] containing MA were detected, with the other acyl moiety generally being saturated fatty acid [26, 64]. Because unsaturated fatty acids are preferentially combined with the *sn*-2-position of phospholipids and diacylglycerol, the diacylglycerol is likely derivatized by MA at the *sn*-2-position, in which case glycerol 2-MA would be formed. This compound was recently detected in human plasma [65]. It is unknown whether the bioactivity of glycerol 2-MA is similar to that of 2-AG.

#### Relation of MA to Diseases

MA is synthesized endogenously, is distributed widely, is a competitor of other PUFAs and is converted to various unique lipid mediators in the body, all of which suggest the physiological and pathological importance of MA. MA metabolism and the activities of MA-derived mediators appear intertwined with ARA metabolism and the systemic regulation of ARA. The relation of MA to various diseases is described below.

#### Inflammation

Many lipid mediators are involved in inflammation and repair. The relation between MA and general inflammation was among the first to be investigated. Dietary supplementation of male Wistar rats with MA increased the concentration of MA in neutrophils and inhibited A23187-stimulated LTB_4_ synthesis in these cells, but did not inhibit the synthesis of two other products of 5-LOX metabolism, 5-HETE and the all-*trans* isomers of LTB_4_ [32, 66]. The authors of that study therefore considered that the effects of MA supplementation resulted from the inhibition of LTA hydrolase. Leukotriene B_4_ synthesis was inhibited in neutrophils from patients with EFA deficiency, and an in vitro experiment suggested that MA is a more potent inhibitor than EPA [67]. Dietary supplementation with MA also increased the content of MA in the phospholipids of peritoneal exudate cells in mice and suppressed the generation of platelet-activating factor in peritoneal cells stimulated by zymosan. The suppression effect of MA was comparable to that of DHA [33]. That study showed that MA supplementation attenuates liver injury induced by galactosamine/lipopolysaccharide in mice. The synthesis of LTB_4_ and LTE_4_ in peritoneal cells was suppressed in MA-fed mice whereas that of PGE_2_ or 6-keto PGE_1α_ was not [34]. These results suggest that MA may inhibit the 5-LOX pathway but not the COX pathway in vivo. However, as described above, the concentration of MA increased in RAW 264.7 cells with increasing culture time and the production of PGD_2_, PGE_2_ and TXA_2_ decreased as LTC_4_ and LTB_4_ decreased [26]. Further studies are needed to clarify the effects of MA on the COX pathway and on the total inflammation phenotype.

#### Cancer

An apparent correlation between cancer and PUFAs, and especially between cancer and n-3 PUFAs in fish, has been actively studied. The World Cancer Research Fund reviewed various cancer risks in 2018 and concluded that there is limited evidence suggesting that consumption of fish decreases the risk of liver cancer and colorectal cancer [68]. Many lipid mediators are involved in the process of cancer, leading to studies on the relation between MA and cancer.

Carcinogenesis is believed to be a sequential multistep process, i.e., DNA damage (initiation phase), enhanced cell proliferation (promotion phase), and metastasis (progression phase). MA inhibits expression of the cell-cell adhesion molecule, E-cadherin, in human squamous cell carcinoma in vitro, affecting metastasis [69]. Another study indicated that MA affects E-cadherin expression and cell proliferation differently, depending on the cell-line (urothelium T-24, breast MCF-7 and colon HRT-18 cells) [21]. Angiogenesis is important for cancer proliferation and metastasis. The content of MA is high in avascular cartilage, as described above, leading to studies on the effect of MA on angiogenesis. Angiogenesis is inhibited by the addition of MA to human umbilical vein endothelial cells [70]. Pathological angiogenesis was recently reported to promote MA accumulation in the retina [71].

The most advanced studies have been conducted on breast cancer. MA suppresses mammary cancers by suppressing cell proliferation, but does not accelerate cell death. MA administration inhibits the growth of KPL-1 human breast cancer cells in vitro and in vivo [35]. The levels of vascular endothelial growth factor receptor (VEGFR) 1 and VEGFR2 decrease upon treatment with MA. VEGF, VEGFR1 and VEGFR2 expression co-localize in KPL-1 cells, indicating that cell growth suppression involves VEGF signaling directly to KPL-1 cells by an autocrine process, although MA apparently does not influence angiogenesis.

The initiation and promotion phases of mammary carcinogenesis in *N*-methyl-*N*-nitrosourea-induced cancer model in rats were also suppressed upon the administration of MA [36]. On the other hand, MA administration did not suppress 7,12‑dimethylbenz[a]anthracene‑induced breast cancer in rats [72]. Different results depending on the carcinogens were reported. With regard to epidemiological data, a nested case-control study reported that the MA composition in plasma total lipids was inversely associated with overall cancer risk and breast cancer risk [73]. Beneficial outcomes following the consumption of omega-9 fatty acids, including MA, for cancer management have been reviewed [74].

#### Dermatitis

Dermatitis is a typical symptom of EFA deficiency [3, 4] and thus the relation between MA and dermatitis has attracted attention. Topical application of MA to the skin of hairless mice causes scaly dermatitis with hyperplasia [75], which formerly led to MA being considered a cause of dermatitis. However, it was recently shown that the abnormality in the epidermal permeability barrier in patients with EFA deficiency is due to the replacement of linoleic acid with oleic acid in *O*-acylsphingolipids [76], suggesting that MA may not necessarily be an exacerbating factor. It was recently reported that dietary coconut oil ameliorated skin contact hypersensitivity through MA production in mice [77]. Intraperitoneal injection of MA inhibits contact hypersensitivity and reduces the number of neutrophils infiltrating the skin, and inhibits the directional migration of neutrophils by inhibiting filamentous actin polymerization and leukotriene B_4_ production. MA inhibits retinol-induced irritant contact dermatitis via PPARα [78], inhibits p38 mitogen-activated protein kinase phosphorylation and prevents both keratinocyte hyperproliferation and the gene expression of neutrophil chemoattractants.

#### Cystic fibrosis (CF)

CF is an autosomal recessive disease caused by mutations in the CF transmembrane conductance regulator gene, which encodes a chloride and bicarbonate channel expressed in the apical membrane of epithelial cells and affects pulmonary, endocrine, gastrointestinal, pancreatic, biliary and reproductive systems [79]. These mutations are believed to have no direct relation to fatty acid metabolism, but defective essential fatty acid metabolism is observed in patients with CF [80]. An increased content of MA and a decrease in EFAs, such as LA, ALA, ARA and DHA, has been observed in the serum of CF patients [81–83]. However, these fatty acid profiles differ from those of typical EFA deficiency, indicating that LA and ALA are slightly decreased but still adequate compared with EFA deficiency. Genotype and sex may have some correlation with EFA status in CF patients [84]. An attempt to reduce morbidity and mortality in CF patients involved 23 randomized controlled trials to compare omega-3 fatty acid supplements with placebo and showed limited benefits with relatively few adverse effects [85].

#### Other diseases

MA is incorporated into platelets [86] and endothelial cells [87]. MA increases the response of platelets to all aggregation agents studied when added simultaneously with the agent [88]. MA suppresses osteoblastic activity in the mouse osteoblast cell line MC3T3-E1 and in goldfish scales [89] and was not associated with risk of posterior longitudinal ligament ossification in a case-control study [90]. Beneficial effects of MA on experimental bowel lesions have been reported [91]. The MA content is low in the serum of phenylketonuria patients [13], and high in the phospholipids in the cerebral cortex of Reelin-deficient mice [21].

However, MA supplementation does not rescue rats from cataract and retinal degeneration induced by *N*-methyl-*N*-nitrosourea [37]. Higher plasma MA levels are associated with fibrosis stage 3–4 of nonalcoholic fatty liver disease [92].

The strength of this study lies in its comprehensive review of MA on biosynthesis, presence, metabolism and physiological and pathological roles. While previous studies have reviewed passive MA formation in response to EFA deficiency, the present review emphasizes the active roles of MA and presents a thorough collection of studies on MA-derived lipid mediators from the 1980s to the present day.

The limitation of this study is its narrative approach, which may cause oversights or biases. Furthermore, the discussion of pathological roles is not entirely satisfactory due to the limited number of supporting studies available. However, inflammatory diseases, cancers, and dermatitis, as demonstrated here, are intensively influenced by lipid mediators, such as PG and LT, which act as both causative and regulatory factors. Recent research developments regarding the impact of MA on these conditions encourage a greater understanding of its potential contributions to therapy. Further studies are needed to explore the pathological roles of MA.

## Conclusion

This review summarizes the biosynthesis, presence, metabolism and physiological roles of MA, and its role in various diseases. MA is synthesized *de novo* in the body and is present not only in conditions of EFA deficiency but also in major normal tissues. An increasing number of studies have reported the relation between MA and diseases, such as inflammation, cancer, dermatitis and cystic fibrosis, and epidemiological data have been reported recently.

### Future perspective

It is expected that further studies directed towards understanding the role of MA, especially to address two points. The first is to clarify the extensive presence of MA physiologically. As described in this review, MA can be synthesized due to topical or temporal EFA deficiency, such as in avascular tissues, even in an EFA-sufficient status. The widespread distribution of MA in tissues will be established soon using new high-resolution techniques. MA is often produced in cultured cells, depending on the experimental conditions. Because it has various unique properties, it is important to determine the fatty acid profile of cultured cells.

The second point is to conduct studies on MA-derived lipid mediators. About forty years ago, MA attracted attention as a natural ARA analogue and was studied extensively but this information is inadequate and out of date. An unknown MA-derived lipid mediator or physiological role perhaps remains to be discovered. MA is an endogenous PUFA, and its various functions may result from MA-derived lipid mediators.

MA tends to be overlooked compared to other n-6 or n-3 PUFAs, but it is widely distributed in tissues and may have various physiological and pathological roles. Further studies on MA will likely improve our understanding of fatty acid metabolism.

## Data Availability

Not applicable.

## Abbreviations

ALA: α-linolenic acid
ARA: Arachidonic acid
CF: Cystic fibrosis
COX: Cyclooxygenase
DGLA: Dihomo-γ-linolenic acid
DHA: Docosahexaenoic acid
DTA: Docosatrienoic acid
EFA: Essential fatty acid
EPA: Eicosapentaenoic acid
HETE: Hydroxyeicosatetraenoic acid
HETrE: Hydroxyeicosatrienoic acid
HPETE: Hydroperoxyeicosatetraenoic acid
LA: Linoleic acid
LOX: Lipoxygenase
LT: Leukotriene
LX: Lipoxins
5-oxo-ETE: 5-oxo-eicosatetraenoic acid
OA: Oleic acid
MA: Mead acid
PG: Prostaglandin
PPAR: Peroxisome proliferator-activated receptor
PUFA: Polyunsaturated fatty acids
VEGF: Vascular endothelial growth factor
